# The Fly as the Main Distributor of Typhoid Fever*Read at the meeting of Seventh Councilor (Austin) District Medical Society, September 23, 1909.

**Published:** 1909-11

**Authors:** Addison L. Lincecum

**Affiliations:** Louise, Texas


For Texas Medical Journal.

### The Fly as the Main Distributor of Typhoid Fever.*

BY ADDISON L. LINCECUM, M. D., LOUISE, TEXAS.

That the common house fly is the most frequent carrier of typhoid infection I have no doubt; however, there is a strong impression among the profession that water is the main carrier, and it has been for many years practically accepted as the correct theory that polluted water was the only common distributor of typhoid infection, therefore we will discuss each topic in a casual manner in this paper, and discuss it more thoroughly in the discussion of the paper by the members of your society.

Water pollution with typhoid fever germs is the least feasible of all theories in my estimation. I do not deny the fact that recently infected water is dangerous to public health, but the typhoid bacilli will live and remain potent only a short time in water, while in dust and moist substances it will live and remain active for long periods (Shropshire). However, we have records of epidemics said to have originated from a case of typhoid fever on the head of a stream in the winter, and in the spring when the snow melted and the streams became polluted with the excreta from the case, the people living many miles below the nidus of infection developed an epidemic of typhoid fever.

Human carriers of typhoid bacilli for long periods after having had the disease are now frequently found, "Typhoid Mary of New York," for illustration. The persistence of typhoid bacilli in the human organism, especially in the biliary passages, without producing symptoms cause persons having had typhoid fever to become a constant source of danger to their fellowmen. E. Huggenberg (*Correspondez-blatt f. Schweizer Aerzte,* Oct. 1, 1908) reports a small epidemic which was traced to a woman who had had typhoid fever thirty-one years before, and since had been well. Repeated examinations of her stools showed the presence of bacillus of Eberth. In the course of thirty-one years, thirteen cases of

---

*Read at the meeting of Seventh Councilor (Austin) District Medical Society, September 23, 1909.

typhoid had occurred in the same house, and eleven of them in the same family. J. A. Pringle refers to a case reported by Branthwaite, in which the cases occurred in groups in an inebriate asylum, and were traced to a dairy maid who had typhoid fever six years previous.

The fact that these cases occurred intermittently, in groups, Dr. Branthwaite explains by pointing out the fact that the bacilli were only present at irregular intervals in the excreta. This point of intermittent occurrence of the bacillus in the stools is important and plainly points out our duty: "Make frequent examination of the stools." (*Dublin Jour. Med. Sci.*, September, 1908.)

Kossel (*Deutsch Med. Wchusch.*, September 26, 1907), Ledingham (*British Med. Jour.*, January 4, 1908), and Dean (*British Med. Jour.*, March 7, 1908), all report similar cases. Chalmers reports five cases contracted from one woman directly, and the rest through the agency of infected milk. In none of these cases was the urine shown to be the source of infection. The bladder and kidneys do not seem to possess the same facilities for becoming a permanent nidus for the germ as do the liver and gall bladder. Pringle says that women appear to become carriers more frequently than men in the proportion of three to one. Doerr (*Centr. f. Bakt. Orig.*, 1905) reports a case in which the bacilli were excreted from the bowels for forty-two years after the fever. These cases I will use as illustrations as to how the fly gets the infection it carries to the intended victim.

The typhoid fly is the commonest of all the flies that are found around human habitation. It is a medium-sized fly, grayish in color and as friendly as a candidate for nomination for Governor, before the primary election. Its mouth parts are widened at the lower extremity with two small openings constructed for sucking liquids and semi-liquid substances. The female fly oviposits preferably upon horse manure, human feces, and in fact upon any filth that may attract them. These eggs hatch in eight to twelve hours and become larvae, which live five days and become pupae, which live five days and the full grown fly comes forth on its mission of scattering disease germs. I wish to call your attention to the peculiar fitness of the fly for this mission. Its entire body is covered with a short growth of scales resembling bristles; its legs are covered with the same growth, except larger; its feet are supplied with two pads which are covered and fringed with bristles; to all of these filth and infectious material adheres. The fly has the most varied appetite of any living thing. It not only feeds on the most delicate and tempting foods, but it goes to the other extreme—it feeds upon the filthiest of matter, human feces.

The question arises, how can the fly take the infection of typhoid to man? There are four methods by which it accomplishes its mission. All of them are mechanical, if you please.

1. By walking over infected material, getting it smeared over its feet and body, then taking it to the food and drink of man.

2. By becoming infected with typhoid fever bacillus and being ingested by man.

3. By feeding on the germs of typhoid and passing them in its feces on the food of man.

4. By being infected and dying and being disseminated as dust and being ingested by man.

Proof that the fly carries typhoid fever:

Dr. V. C. Vaughan and associates first called the attention of the War Department to the fact that typhoid fever was carried by flies, during the Spanish-American war.

Dr. Dutton in a paper read before the A. M. A., 1909, says: "I noticed that typhoid fever epidemics spread in the direction of the prevailing winds, during the epidemic in the army camps in Florida; also noticed that the flies around the mess sheds had lime on their feet and bellies from the garbage dumps and the latrines, and that the epidemic began to subside as soon as the weather became cool enough to disable the flies."

Dr. Thos. S. Anderson, of Pittsburg, in a recent paper confirms the spread of typhoid with the prevailing wind, and that the flies traveled with the wind.

The Merchants' Association sent inquiries to all the investigators, health officers, and well known sanitarians in the United States, and below we give you the results:

| Typhoid fly. | Milk. | Water. | Non-committal. |
|---|---|---|---|
| 49 | 10 | 15 | 4 |

One interesting report they received I will relate in detail, epidemic in Washington, D. C. Total cases 5,808, divided as follows:

| Causes— | No. of Cases. | Per cent. |
|---|---|---|
| Contact cases | 366 | 7.51 |
| Due to milk | 135 | 2.77 |
| Unknown origin | 4,367 | 89.72 |

With the evidence in hand that the human body may retain a nidus of typhoid bacilli and excrete the same at irregular intervals, even as long as forty-two years, and that this excreta, infected as it is, furnishes an incubator for the flies to propagate in and furnishes food for the young flies immediately after they are hatched, considering their natural physical fitness to carry this infection around on their feet and bodies, as well as excrete the germs in their feces, combined with their natural fondness for human companionship, especially at meal time, it stands to reason that they play a most important part in the transmission of typhoid.

Let me draw in your imagination a picture, one which no doubt many of you have already seen in reality. We will suppose a case of typhoid fever in a farm house, unscreened, with a shallow well about thirty feet away, a horse lot and an open closet within 100 feet, flies present in countless numbers. The vessel containing the excreta of the patient is taken from the room, its contents carefully emptied as near the well as permissible, a little water is dashed into the vessel and it is emptied right at the well, the flies swarm over the excreta, and then over the food and the whole family takes typhoid. The attending physician puts on a long face and says: "Bill, your well is contaminated, and your whole family have taken typhoid fever from the water."

The three most dangerous things in any community is "a typhoid mary," an open closet, and an abundance of flies.

Your efficient secretary has during the past few months been endeavoring to procure data on the origin of typhoid fever, whether it is due to water or to flies. He having courteously allowed me the use of the data in his hands, I will read it to you:

"The answers to the questions sent out to all physicians reporting a death from typhoid have come in. Only thirty-four physicians responded. The total number of cases of typhoid reported on is 97. Of these, the house was screened in only 11, although 40 of the cases occurred in city practice. A horse lot existed close by in 86 cases. The house fly was noticeably prevalent in 71 cases. In only 8 cases was the house fly noticeably rare. Of course, we do not blame the house fly with every single case, but his presence is so uniform that we strongly suspect him. We suspect him especially because there were 57 open closets against 22 sanitary closets. Water of all kinds was used, although the shallow well predominated." (Table omitted.)

Gentlemen, with the evidence I have attempted to give you in this brief paper, it seems to me that the fly is proven beyond the peradventure of a reasonable doubt as the main distributor of typhoid fever. However, bear in mind this fact: It is dangerous to accept the fly alone as the sole distributor of this disease, for you well know where we look at a given point in the origin of a disease, we may be neglecting some very important factor. I have come some three hundred miles to hear this paper discussed, and I want every man here to discuss freely and without any sense of restraint, for the real value of a paper is the discussion. If you disagree with the writer on any point, say so, and tell why. You may bring out a new point, or some feature that has been neglected in writing the article.

The paper brought out an animated discussion. The verdict was: "Mr. Fly is guilty as charged in the indictment." A vote of thanks was given Dr. Lincecum.

---

MEDICINAL TREATMENT OF RICKETS.—Colbeck gives the following medicinal treatment for rickets (*The Practitioner*, September, 1909):

℞ Syrup of ferrous iodide........................℥i
Cod liver oil...........................ad ℥viii

M. et Sig.: Two teaspoonfuls to be taken three times a day after food.

℞ Phosphorated oil..........................Mxvi
Cod liver oil...............................℥iv

M. et Sig.: One teaspoonful to be taken three times a day after meals.

℞ Cod liver oil...............................ʒv
Syrup of calcium lactophosphate..............℥iss
Lime water..................................℥iss

M. et Sig.: One teaspoonful to be taken three times a day after meals.

℞ Sodium hypophosphite.....................gr. v
Calcium hypophosphite.......................gr. v
Iron hypophosphite..........................gr. ii
Sodium bicarbonate..........................gr. v
Spirit of chloroform........................Mxv
Camphor water..........................q. s. ad ℥i

M. et Sig.: One teaspoonful to one tablespoonful to be taken three times a day.

℞ Calcium hypophosphite....................gr. ii
Syrup of iron phosphate.....................ʒss
Simple syrup, q. s., ad.....................ʒi

M. et Sig.: OOne teaspoonful to be taken three times a day.

